# Survival of patients receiving systematic therapy for metachronous or synchronous metastatic renal cell carcinoma: a retrospective analysis

**DOI:** 10.1186/s12885-019-5900-1

**Published:** 2019-07-15

**Authors:** Sung Han Kim, Dong-eun Lee, Boram Park, Jungnam Joo, Jae Young Joung, Ho Kyung Seo, Kang Hyun Lee, Jinsoo Chung

**Affiliations:** 1 Department of Urology, Center for Prostate Cancer, National Cancer Center, 323 Ilsan-ro, Ilsandong-gu, Goyang-si, Gyeonggi-do 10408 Republic of Korea; 2 Biometrics Research Branch, Division of Cancer Epidemiology and Prevention, Research Institute and Hospital of National Cancer Center, Goyang, South Korea

**Keywords:** Renal cell carcinoma, Metastasis, Synchronous, Metachronous, Prognosis, Immunotherapy, Targeted therapy

## Abstract

**Background:**

The differences in progression-free survival (PFS) and cancer-specific survival (CSS) of metastatic renal cell carcinoma (mRCC) patients according to treatment, type of metastasis, and Heng criteria risk are unclear. In this study, we compared survival according to various such parameters.

**Methods:**

Between 2000 to 2014, 214 mRCC patients, of whom 171 (79.9%) were intermediate-risk and 43 (20.1%) were poor-risk, were retrospectively selected; 126 (58.9%) patients were treated with immunotherapy (IT) and 88 (41.1%) with targeted therapy (TT). Moreover, 144 patients had synchronous mRCCs (67.3%, SM) and 70 had metachronous mRCCs (32.7%, MM). The Kaplan-Meier method and log-rank test were used to compare progression-free survival (PFS) and CSS.

**Results:**

During a median 4.2 (1.0–70.4) months of systemic treatment and 98.3 (4.8–147.6) months of follow-up, the median PFS and CSS were 4.7 (95% confidence interval [CI]: 3.8–5.5) and 13.8 (95% CI, 9.8–18.3) months, respectively. The PFS and CSS were significantly better in the MM (5.9 and 21.3 months) and intermediate-risk groups (5.2 and 18.3 months) than those in the SM (4.4 and 9.6 months) and poor-risk groups (2.7 and 5.8 months), respectively (*p* < 0.05). Further stratification showed that TT produced significantly better PFS than IT in intermediate-risk patients with SM and a treatment-free interval (TFI) < 1 year, and in those with MM with a TFI ≥1 year (*p* < 0.05). There were no differences in survival outcomes according to various other subgroup stratifications (*p* > 0.05).

**Conclusion:**

Dividing patients into specific subcategories helps to better predict therapeutic outcomes.

**Electronic supplementary material:**

The online version of this article (10.1186/s12885-019-5900-1) contains supplementary material, which is available to authorized users.

## Background

The standard systemic therapy for metastatic renal cell carcinoma (mRCC) has recently been changed to targeted therapy (TT). TT produces improved prognosis, with an observed median cancer-specific survival (CSS) of 29.5 months, and has markedly extended progression-free survival (PFS) intervals [1]. Furthermore, TT is relatively well tolerated compared to immunotherapy (IT) with cytokines [2], which was the mainstay systemic treatment in the past decades [3]. Despite poor 5-year survival rates of approximately 10%, IT had an advantage of significantly improving overall survival (OS), with some patients achieving 10-year persistent complete remission rates of 7–10% [4, 5]. Conversely, whether TT prolongs OS has not yet been determined [4]. The heterogeneous characteristics of mRCC patients, as well as the heterotrophic characteristics of their tumors, makes the prediction of therapeutic outcomes challenging. Therefore, dividing patients into subcategories ought to be considered more thoroughly to derive more reliable predictive prognostic factors.

mRCCs are categorized into synchronous mRCC (SM), which involves approximately one-third of newly diagnosed patients; and metachronous mRCC, (MM), that has a prevalence rate of 30–40% among localized RCC patients and involves disease progression to metastasis after a certain interval of time has elapsed since curative surgery. MM and SM have different pathophysiologies and metabolisms; furthermore, the heterotrophic and pleomorphic characteristics of RCC result in unpredictable and diverse responses to systemic therapy. Moreover, the microenvironments and tumor activities of primary vs. metastatic tumor lesions, as well as their underlying tumor burdens, are different [3, 6]. However, the TT treatment guidelines for MM and SM are similar, and not many studies have addressed the differential prognoses and comparative responses between TT and IT according to the tumors’ metastatic types and prognostic risk groups. Such clinical data regarding survival outcomes are required for clinicians to understand the patients’ prognoses and to devise effective treatment strategies. Therefore, this retrospective study analyzed the PFS and CSS according to first-line systemic therapy, as well as the survival rates of patients with MM or SM mRCCs treated with either TT or IT at a single institution. Furthermore, prognostic outcomes according to the types of metastases were compared with respect to the Heng risk model and treatment-free intervals (TFIs).

## Methods

### Ethics statement

Following approval of this retrospective study by the Institutional Review Board of the National Cancer Center (IRB No. NCC2016–0263), the IRB waived the written informed consent requirement. All patient data were anonymized and de-identified prior to our analysis. All study protocols were performed in accordance with the ethical tenets of the Declaration of Helsinki.

### Patients’ criteria and evaluating tools

The medical records of 214 mRCC patients treated between 2000 and 2014, including 171 (79.9%) intermediate-risk and 43 (20.1%) poor-risk patients according to the Heng criteria, were retrospectively reviewed. Patients with the following characteristics were excluded from the analysis: those with incomplete follow-up medical records (records beyond the prospectively recorded National Center Center RCC registry), patients who refused systemic therapies after receiving an explanation of the possible adverse events, patients who stopped receiving medications because of the associated financial expenses, patients for whom no therapeutic effects were expected because of poor clinical statuses involving multiple underlying diseases and severe tumor burdens, patients with missing data for all of the risk factors included in the analysis, patients with missing treatment records, patients under 20 years of age, patients belonging to the favorable risk group according to the Heng risk criteria, and patients who developed progression within 1 month of treatment (Additional file 1: Figure S1). There were 144 SM patients (67.3%) and 70 MM patients (32.7%); 126 patients were treated with IT and 88 were treated with TT. All mRCC patients underwent a complete evaluation after every 1–4 cycles (6–12 weeks) of IT and every 2 cycles of TT (12 weeks). The follow-up protocol that included laboratory and imaging evaluations was previously described in detail [7]. Treatment continued until disease progression was detected. Patients were further stratified into TFI < 1 year vs. TFI ≥1 year groups. The TFI was defined as the time from the diagnosis of disease to the start of systemic treatment [8–10]

The International Metastatic Renal Cell Carcinoma Database Consortium risk criteria (also known as the Heng criteria) [8] for prognostic risk stratification, the Response Evaluation Criteria in Solid Tumors v1.1 for therapeutic responsive evaluation to systemic therapy [11], and the Fuhrman nuclear grade [12] and TNM stages for pathological RCC evaluation [13] were used.

### Treatment regimens

The choice of systemic agent (IT or TT) was at the discretion of the treating urologist (J.C.) according to each patient’s pathology and coverage by the National Health Insurance System, as described previously [7]. Combination IT comprised of subcutaneous recombinant human interleukin (IL)-2 (Proleukin, Chiron V.B.), recombinant human interferon (IFN)-α (IFN-alpha-2a, Roferon-A, Roche), intravenous 5-fluorouracil (5-FU, JW Pharm), and vinblastine (vinblastine, United Pharm, Korea). Triple or quadruple regimens were administered according to our previous cited regimen [14].

All TTs were administered either orally or intravenously with the recommended regimen in the National Comprehensive Cancer Network guidelines, version 2.2016 (available at http://www.nccn.org/patients for patients). First-line TT comprised sunitinib, sorafenib, pazopanib, or temsirolimus; sequential TT included sunitinib, sorafenib, pazopanib, temsirolimus, bevacizumab, everolimus, or axitinib. Targeted-agent regimens were described previously [7, 15].

### Statistical analysis

The baseline characteristics are summarized as frequency with percentage for categorical variables and median with range (min-max) for continuous variables. The differences between SM and MM were assessed using the Wilcoxon rank-sum test and chi-square test (or Fisher’s exact test). The date of the most recent hospital visit was used as the date of last follow-up. The PFS duration was defined as the time between first-line systemic therapy and disease progression. The CSS duration was defined as the time between first-line systemic therapy and death from cancer or alive. Follow-up durations were estimated using the reverse Kaplan-Meier method, in which being alive is treated as the event of interest and deaths are censored. The estimated survival curves were computed using the Kaplan-Meier method. Survival curves according to systemic treatment and Heng risk groups were compared using the log-rank test. Cox proportional hazards models were used to analyze subgroups of the patients with mRCC. To identify factors associated with prognosis, univariable Cox models of PFS and CSS were applied to the variables included in the Heng risk model. Variables that had significant (*p* < 0.05) associations with PFS or CSS in the univariable analyses were included in multivariable Cox models. A backward variable selection method was then applied to the included variables with an elimination *p*-value criterion of 0.05. *P*-values less than 0.05 were considered statistically significant. All statistical analyses were performed using SAS (version 9.3; SAS Institute Inc., Cary, NC, USA) and R (version 3.3.2) software.

## Results

Following a median 4.2 (range: 1.0–70.4) months of systemic treatment and 98.3 (range: 4.8–147.6) months of follow-up, only 30 patients (14.0%) were alive at the end of the study. The median PFS and CSS were 4.7 (95% confidence interval: 3.8–5.5) months and 13.8 (95% confidence interval: 9.8–18.3) months, respectively. The patients’ characteristics are summarized in Table 1. The gender ratio, nephrectomy rates, TFIs, Heng risk groups, clinical T and N stages, clear cell and non-clear cell histologies, follow-up durations, PFS, and CSS were significantly different between the SM and MM groups (*p* < 0.05, Table 2).

**Table 1:** Baseline characteristics table (*N* = 214)

		N (%) or Median(min-max)
Age (years)		58 (26–81)
Gender	Male	166 (77.6)
	Female	48 (22.4)
Nephrectomy	No	90 (42.1)
	Yes	121 (56.5)
	Unknown	3 (1.4)
Treatment free interval	≥1 yr	56 (26.2)
	< 1 yr	158 (73.8)
Anemia	Normal	44 (20.6)
	Hb < 13.5/12.0 (M/F)	170 (79.4)
Hypercalcemia	Normal	180 (84.1)
	> 10 mg/dL or 2.5 mmol/L	28 (13.1)
	Unknown	6 (2.8)
Neutrophilia	Normal	174 (81.3)
	< 1500 or > 7500	33 (15.4)
	Unknown	7 (3.3)
Elevated LDH	Normal	96 (44.9)
	>×1.5ULN	49 (22.9)
	unknown	69 (32.2)
KPS	> 80	204 (95.3)
	≤ 80	4 (1.9)
	unknown	6 (2.8)
Thrombocytosis	Normal	192 (89.7)
	> 400 K	22 (10.3)
Therapy	Immunotherapy	126 (58.9)
	Target Therapy	88 (41.1)
Heng	Intermediate risk	171 (79.9)
	Poor risk	43 (20.1)
Tumor (T)	T1 - T2	96 (44.9)
	T3 -T4	61 (28.5)
	Unknown	57 (26.6)
Lymph node(N)	N0	54 (25.2)
	N1	45 (21.0)
	Nx	30 (14.0)
	Unknown	85 (39.7)
Metastasis	cM0	27 (12.6)
	cM1	81 (37.9)
	pM1	2 (0.9)
	cMx	3 (1.4)
	Unknown	101 (47.2)
mRCC type	Synchronous mRCC	144 (67.3)
	Metachronous mRCC	70 (32.7)
Fuhrman nuclear grade	G1-G2	41 (19.2)
	G3-G4	101 (47.2)
	Unknown	72 (33.6)
Histology	Clear cell	164 (76.6)
	Non-clear cell	12 (5.6)
	Unknown	38 (17.8)
Treatment duration (Month)		4.2 (1.0–70.4)
Follow-up duration (Month)		98.3 (4.8–147.6)
Progression free survival (Month, median(95%CI))		4.7 (3.8–5.5)
Cancer-specific survival (Month, median(95%CI))		13.8 (9.8–18.3)
Cancer-specific survival status	Censored/ Event	32 (15.0)/ 182 (85.0)
Progression-free survival status	Censored/ Event	21 (9.8)/ 193 (90.1)

**Table 2:** Comparison of baseline characteristics between synchronous (*N* = 144) and metachronous (*N* = 70) mRCC groups

		Synchronous	Metachronous	*P*-value
		(*N* = 144)	(*N* = 70)	
Age (years, min-max)		59.0 (26.0–81.0)	56.0 (33.0–76.0)	0.232^a^
Gender	Male	118 (81.9)	48 (68.6)	0.028^b^
	Female	26 (18.1)	22 (31.4)	
Nephrectomy	Yes	52 (36.6)	69 (100.0)	<.001^b^
Treatment free interval	≥1 yr	8 (5.6)	48 (68.6)	<.001^c^
	< 1 yr	136 (94.4)	22 (31.4)	
Anemia		111 (77.1)	59 (84.3)	0.221^b^
Hypercalcemia		20 (14.4)	8 (11.6)	0.578^b^
Neutrophilia		21 (15.1)	12 (17.7)	0.639^b^
Elevated LDH	>×1.5ULN	39 (38.2)	10 (23.3)	0.082^b^
KPS	> 80	135 (97.1)	69 (100)	0.304^c^
	≤ 80	4 (2.9)	0 (0)	
Thrombocytosis	> 400 K	18 (12.5)	4 (5.7)	0.125^b^
First line therapy	Immunotherapy	89 (61.8)	37 (52.9)	0.212^b^
	Targeted therapy	55 (38.2)	33 (47.1)	
Heng risk	Intermediate	107 (74.3)	64 (91.4)	0.003^b^
	Poor	37 (25.7)	6 (8.6)	
Tumor (T)	T1 - T2	70 (67.3)	26 (49.1)	0.027^b^
	T3 -T4	34 (32.7)	27 (50.9)	
Lymph node(N)	N0	49 (47.1)	5 (20.0)	<.001^b^
	N1	25 (24.0)	20 (80.0)	
	Nx	30 (28.9)	0 (0.0)	
Fuhrman nuclear grade	G1-G2	30 (33)	11 (21.6)	0.151^b^
	G3-G4	61 (67)	40 (78.4)	
Histology	Clear cell	113 (96.6)	51 (86.4)	0.022^c^
	Non-clear cell	4 (3.4)	8 (13.6)	
Treatment duration (Month)		3.4 (1.0–70.4)	5.3 (1.0–62.0)	0.058^a^
Follow-up duration (Month)		81.3 (4.8–141.5)	142.3 (9.4–147.6)	0.005^d^
Progression free survival (Month, median(95%CI))		4.4 (3.1–5.2)	5.9 (4.1–9.5)	0.044^d^
Cancer specific survival (Month, median(95%CI))		9.6 (7.4–13.8)	21.3 (15.1–29.3)	<.001^d^

*KPS* Karnofsky performance status

^a^Wilcoxon rank sum test, ^b^Chi-square test, ^c^Fisher exact test, d: Log-rank test

Significantly better PFS and CSS rates in the MM group (PFS: 5.9 months; CSS: 21.3 months) and in the intermediate-risk group (PFS: 5.2 months; CSS: 18.3 months) were observed than in the SM (PFS: 4.4 months; CSS: 9.6 months) and poor-risk groups (PFS: 2.7 month; CSS: 5.8 months) (*p* < 0.05; Additional file 1: Figure S2). In the intermediate-risk groups, patients with MM showed longer PFS and CSS rates than those with SM (PFS: 6.2 vs. 5.1 months; CSS: 25.2 vs. 13.9 months, respectively). Furthermore, the MM group showed longer PFS and CSS than the SM group (3.7 vs. 2.7 months and 10.2 vs. 5.6 months, respectively) in poor-risk patients (Additional file 1: Figure S2); statistical significance was only achieved for CSS in the intermediate-risk group (*p* = 0.01) (Additional file 1: Figure S3).

**Fig. 1:**
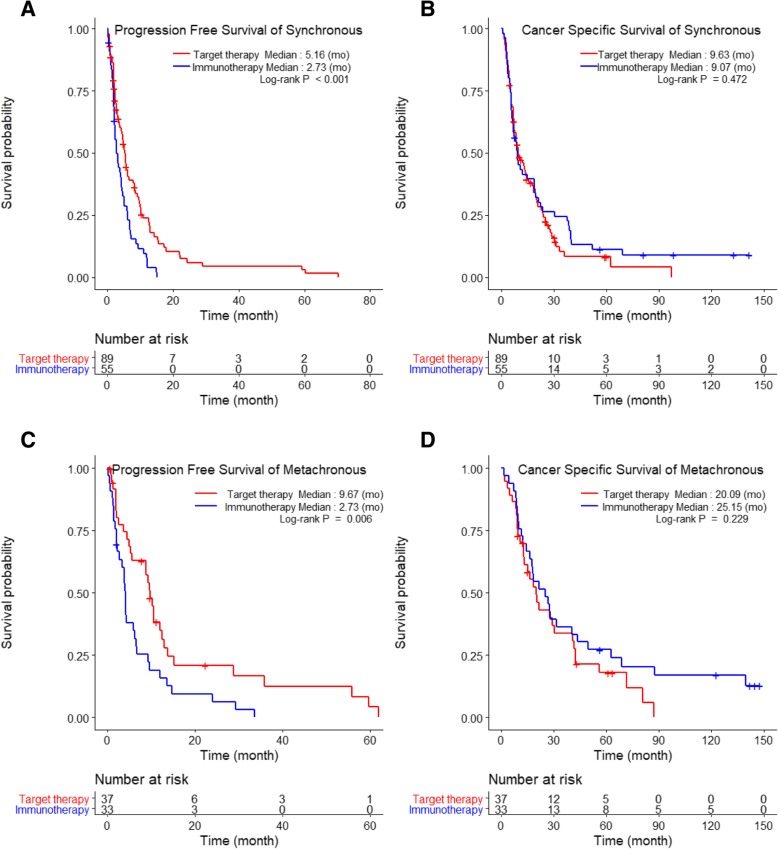
The comparison of Kaplan-Meier curves of progression-free survival and cancer-specific survival between metastatic renal cell carcinoma patients receiving immunotherapy (IT) and targeted therapy (TT) in those with metachronous and synchronous metastases

Among patients with SM, those receiving TT had significantly longer PFS (5.2 months) than those receiving IT (2.7 months, *p* < 0.001; Fig. 1a). Similarly, among patients with MM, those receiving TT (9.7 months) had significantly longer PFS than those receiving IT (4.1 months, *p* = 0.006; Fig. 1c). However, CSS did not differ significantly between SM patients receiving IT (9.1 months) and those receiving TT (9.6 months; *p* = 0.472; Fig. 1b), or between MM patients receiving IT (25.2 months) and those receiving TT (20.1 months; *p* = 0.229; Fig. 1d).

**Fig. 2:**
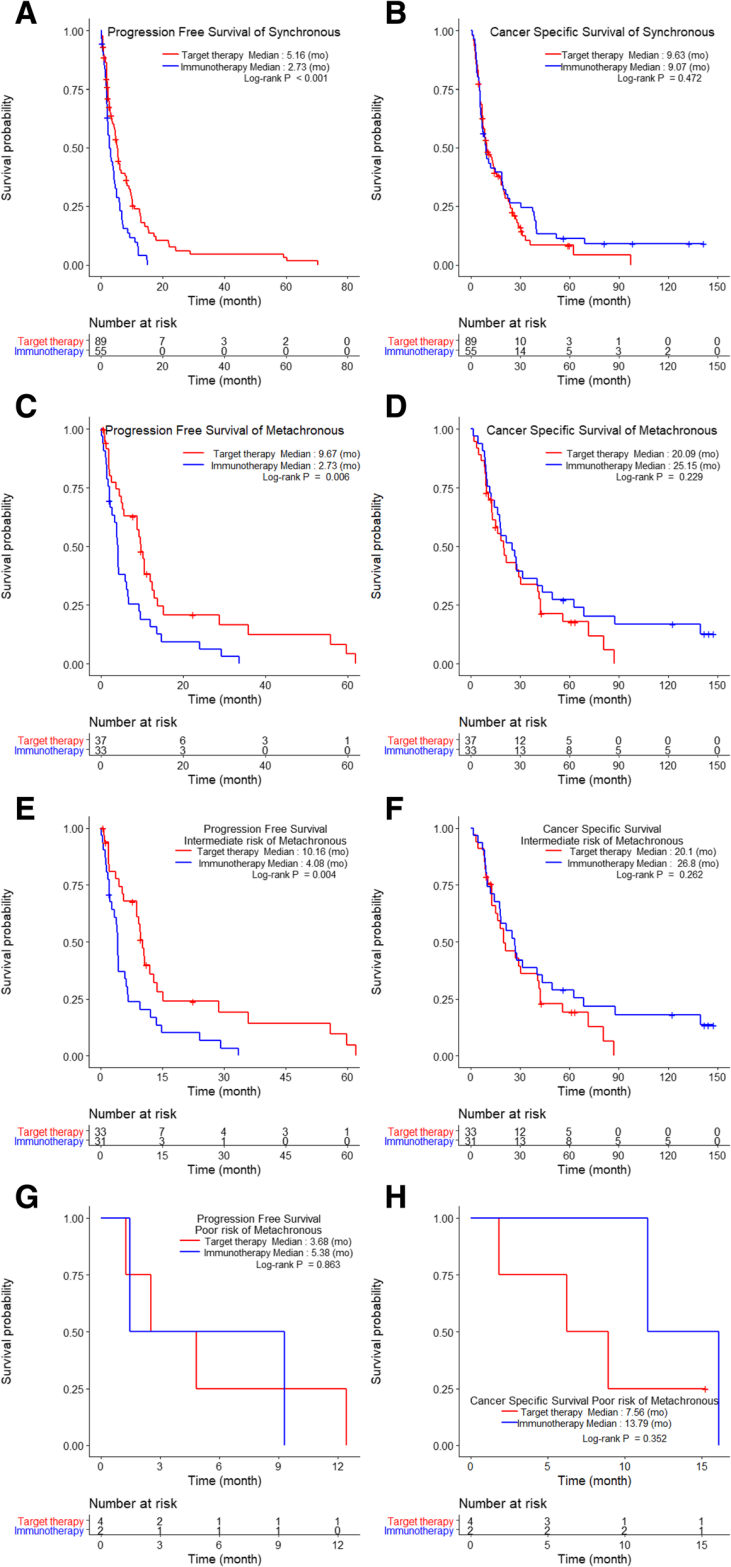
Comparison of Kaplan-Meier curves of progression-free survival and cancer-specific survival of metastatic renal cell carcinoma patients receiving immunotherapy (IT) and targeted therapy (TT) in groups with synchronous metastases (**a**–**d**) and metachronous metastases (**e**–**h**) according to the Heng risk groups

As for the stratified Heng risk groups, only the intermediate-risk group treated with TT had a significantly longer PFS (6.1 months) than that of the IT group (2.6 months) among SM patients (*p* < 0.001, Fig. 2a), although CSS was not significantly different (*p* = 0.67; Fig. 2b). There were no differences in PFS and CSS when comparing IT vs. TT among poor-risk SM patients (*p* = 0.164 for PFS, *p* = 0.083 for CSS; Fig. 2c-d). TT significantly improved PFS (TT: 10.2 months; IT: 4.1 months, *p* = 0.004; Fig. 2e) but not CSS among patients with MM in the intermediate-risk group (*p* = 0.262; Fig. 2f), whereas PFS and CSS were not significantly different when comparing IT vs. TT among poor-risk MM patients (*p* = 0.863 for PFS, *p* = 0.352 for CSS; Fig. 2g-h).

**Fig. 3:**
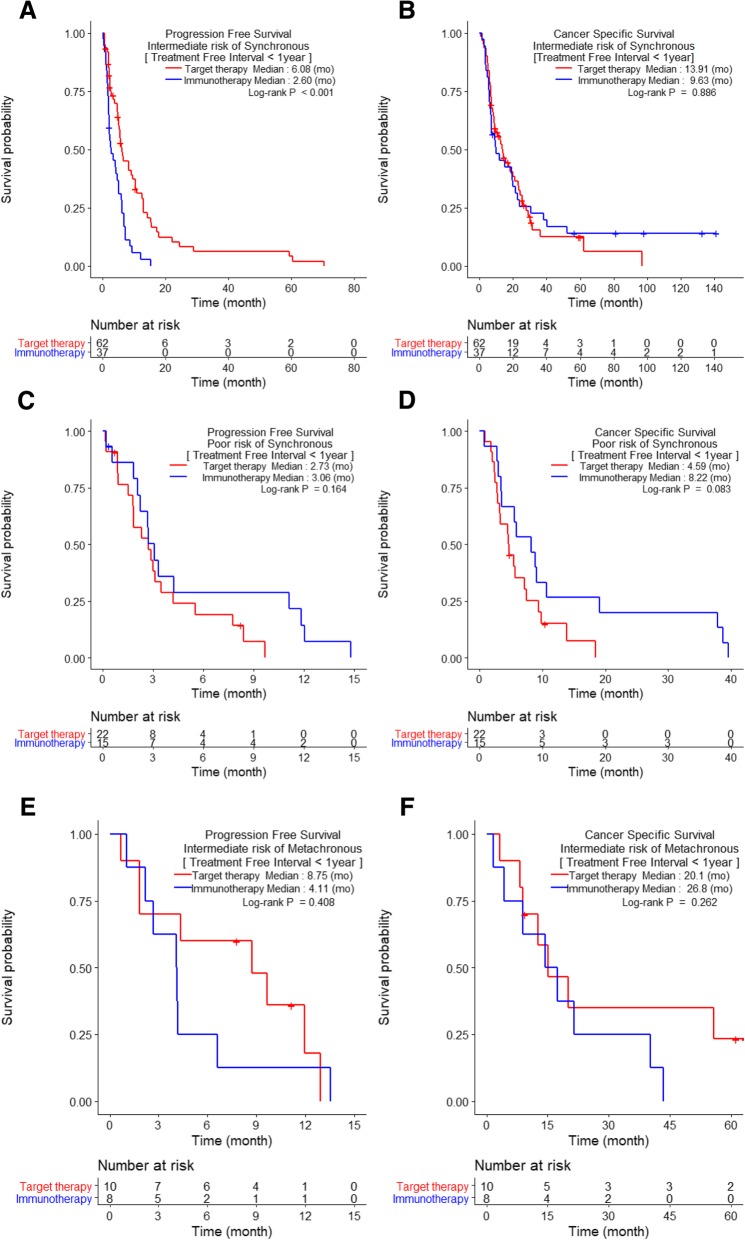
Comparison of Kaplan-Meier curves of progression-free survival and cancer-specific survival of metastatic renal cell carcinoma patients with treatment-free intervals <1 year and with synchronous metastases (**a**–**d**) and metachronous metastases (**e**–**f**) between patients receiving immunotherapy (IT) and targeted therapy (TT) according to the Heng risk groups

**Fig. 4:**
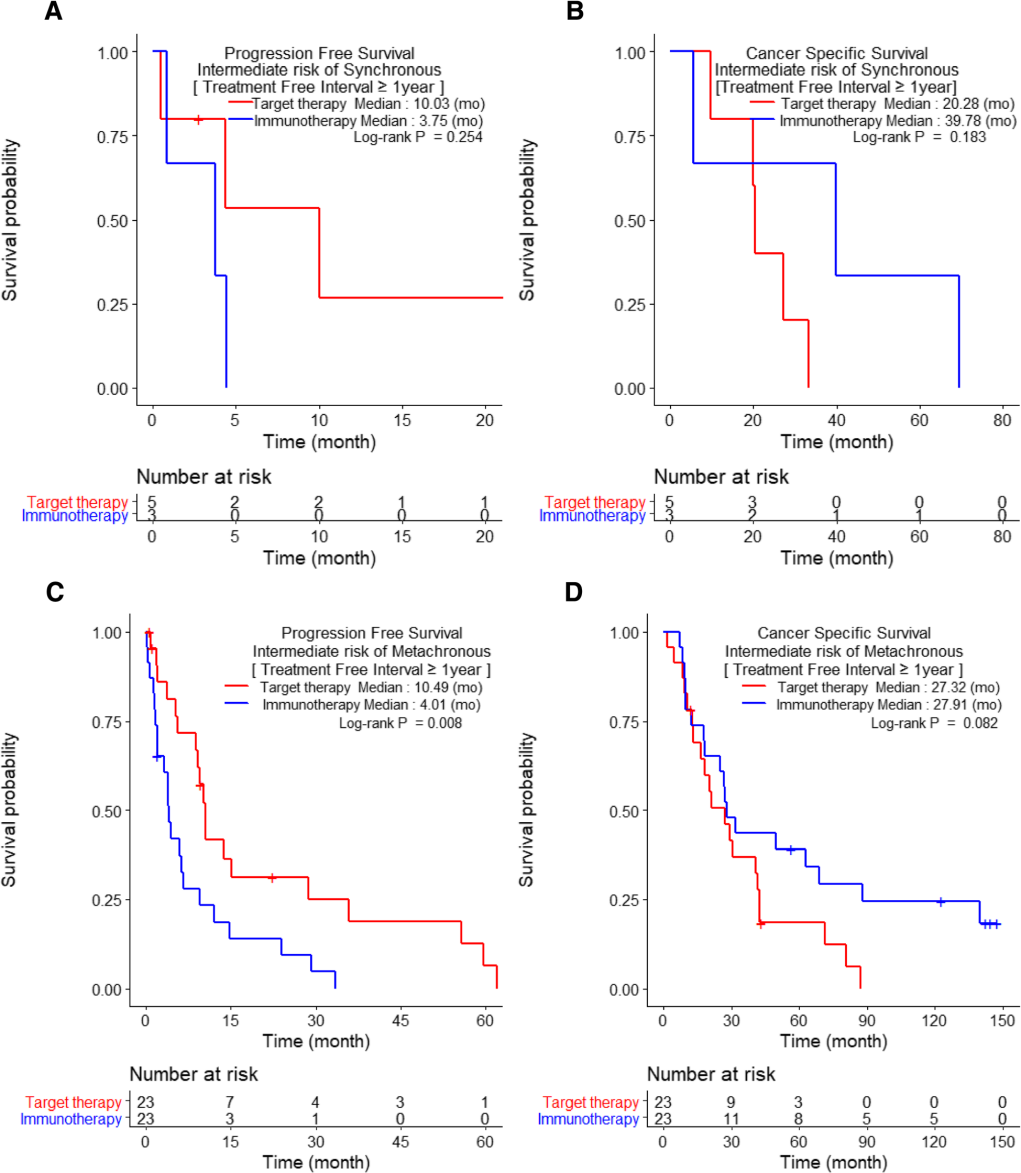
Comparison of Kaplan-Meier curves of progression-free survival and cancer-specific survival of metastatic renal cell carcinoma patients with treatment-free intervals ≥1 year and with synchronous metastases (**a**–**b**) and metachronous metastases (**c**-**d**) between patients receiving immunotherapy (IT) and targeted therapy (TT); shown are intermediate-risk patients according to the Heng criteria

When stratifying patients according to TFI of < 1 year vs. ≥1 year, intermediate-risk patients treated with TT had significantly better PFS than those treated with IT (6.1 months vs. 2.6 months, respectively) among SM patients with TFI < 1 year (*p* < 0.001, Fig. 3a). However, no other significant differences in PFS or CSS were observed between IT- and TT-treated patients with SM or MM and TFI < 1 year (*p* > 0.05, Fig. 3). Among patients with mRCC and TFIs ≥1 year, only intermediate-risk MM patients treated with TT had significantly better PFS (10.5 months) than those treated with IT (4.0 months) (*p* = 0.008; Fig. 4c). The poor-risk patients with TFIs ≥1 year could not be assessed because of their small sample size.

The results of univariable and multivariable Cox proportional hazards analyses of PFS and CSS are summarized in Additional file 1: Table S1. In the univariable analyses of PFS, metastatic type, therapy, TFI, neutrophilia, and elevated LDH were statistically significant prognostic factors. In the multivariable analysis of PFS, therapy (hazard ratio [HR]: 0.537; 95% CI: 0.399–0.724; *p* < 0.001), TFI (HR: 1.639; 95% CI: 1.171–2.293; *p* = 0.004), and neutrophilia (HR: 1.695; 95% CI: 1.141–2.516; *p* = 0.009) were statistically significant factors. In the univariable analyses of CSS, metastatic type, TFI, hypercalcemia, neutrophilia, elevated LDH, and thrombocytopenia were significant prognostic factors. In the multivariable analysis of CSS, TFI (HR: 2.115; 95% CI: 1.484–3.015; *p* < 0.001), hypercalcemia (HR: 1.788; 95% CI: 1.163–2.748; *p* = 0.008), neutrophilia (HR: 1.916; 95% CI: 1.247–2.942; *p* = 0.003), and thrombocytosis (HR: 1.856; 95% CI: 1.092–3.157; *p* = 0.022) were significant factors.

Cox analyses of PFS for the SM and MM subgroups are shown in Table 3. In univariable analyses of the SM subgroup, Heng risk classification, therapy, anemia, neutrophilia, and thrombocytopenia were significant prognostic factors. In the multivariable analysis of the SM subgroup, therapy (HR: 1.696; 95% CI: 1.157–2.486; *p* = 0.0068) and neutrophilia (HR: 2.105; 95% CI: 1.274–3.478; *p* = 0.0037) were significant factors. In univariable analyses of the MM subgroup, therapy was the only significant factor.

**Table 3:** The Cox proportional hazards model of progression-free survival in synchronous and metachronous mRCC group

		Synchronous mRCC (*N* = 144 /EVENT = 130)				Metachronous mRCC (*N* = 70/EVENT = 63)			
		Univariable		Multivariable		Univariable		Multivariable	
		Hazard ratio (95% CI)	*P*-value	Hazard ratio (95% CI)	*P*-value	Hazard ratio (95% CI)	*P*-value	Hazard ratio (95% CI)	*P*-value
Risk group	Intermediate	1				1			
	poor	1.728 (1.156–2.584)	0.0077			1.801 (0.766–4.235)	0.1772		
Therapy	IT	1		1		1		1	
	TT	1.858 (1.288–2.681)	0.0009	1.696 (1.157–2.486)	0.0068	2.019 (1.202–3.39)	0.0079	2.019 (1.202–3.39)	0.0079
Treatment Free interval	≥1 yr	1				1			
	< 1 yr	1.017 (0.473–2.184)	0.9660			1.752 (0.995–3.084)	0.052		
Gender	Male	1				1			
	Female	1.215 (0.777–1.901)	0.3934			1.155 (0.670–1.993)	0.6041		
Age (year)		0.991 (0.975–1.007)	0.2801			0.980 (0.951–1.009)	0.1692		
Anemia	Normal	1				1			
	Hb < 13.5(male)/12.0(Female)	1.724 (1.142–2.600)	0.0095			0.846 (0.427–1.675)	0.6311		
Hypercalcemia	Normal	1				1			
	> 10 mg/dL or 2.5 mmol/L	1.166 (0.705–1.929)	0.5499			0.995 (0.444–2.227)	0.9897		
Neutrophilia	Normal	1		1		1			
	< 1500 or > 7500	2.497 (1.53–4.074)	0.0002	2.105 (1.274–3.478)	0.0037	0.918 (0.464–1.817)	0.806		
Elevated LDH	Normal	1				1			
	1.5 X ULN	1.482 (0.943–2.329)	0.0883			1.491 (0.661–3.365)	0.3357		
KPS	> 80	1							
	≤ 80	2.122 (0.779–5.779)	0.1409						
Thrombocytosis	Normal	1				1			
	> 400 K	1.700 (1.013–2.855)	0.0448			1.054 (0.379–2.932)	0.9199		

Cox analyses of CSS in the SM and MM subgroups are shown in Table 4. In univariable analyses of the SM subgroup, Heng risk classification, hypercalcemia, neutrophilia, and thrombocytopenia were significant prognostic factors. In the multivariable analysis of the SM subgroup, hypercalcemia (HR: 2.164; 95% CI: 1.307–3.584; *p* = 0.0027) and neutrophilia (HR: 2.655; 95% CI: 1.601–4.405; *p* = 0.0002) were significant factors. In univariable analyses of the MM subgroup, Heng risk classification and TFI were significant factors.

**Table 4:** The Cox proportional hazards model of cancer specific survivalin synchronous and metachronous mRCC group

		Synchronous mRCC (*N* = 144 / EVENT = 123)				Metachronous mRCC (*N* = 70 / EVENT = 59)			
		Univariable		Multivariable		Univariable		Multivariable	
		Hazard ratio (95% CI)	*P*-value	Hazard ratio (95% CI)	*P*-value	Hazard ratio (95% CI)	*P*-value	Hazard ratio (95% CI)	*P*-value
Risk group	Intermediate	1				1		1	
	poor	2.377 (1.591–3.553)	<.0001			3.536 (1.332–9.390)	0.0113	3.536 (1.332–9.390)	0.0113
Therapy	IT	1				1			
	TT	0.854 (0.590–1.237)	0.4041			0.725 (0.428–1.228)	0.2312		
Treatment Free interval	≥1 yr	1				1			
	< 1 yr	1.465 (0.714–3.009)	0.2978			1.918 (1.089–3.378)	0.0241		
Gender	Male	1				1			
	Female	1.185 (0.758–1.854)	0.4557			1.313 (0.768–2.246)	0.3200		
Age (year)		1.002 (0.985–1.019)	0.8225			0.986 (0.959–1.013)	0.3126		
Anemia	Normal	1				1			
	Hb < 13.5(male)/12.0(Female)	1.555 (0.977–2.475)	0.0626			0.738 (0.372–1.464)	0.3841		
Hypercalcemia	Normal	1		1		1			
	> 10 mg/dL or 2.5 mmol/L	2.118 (1.297–3.460)	0.0027	2.164 (1.307–3.584)	0.0027	0.948 (0.429–2.098)	0.8956		
Neutrophilia	Normal	1		1		1			
	< 1500 or > 7500	2.595 (1.589–4.238)	0.0001	2.655 (1.601–4.405)	0.0002	1.296 (0.631–2.661)	0.4798		
Elevated LDH	Normal	1				1			
	1.5 X ULN	1.482 (0.943–2.329)	0.0883			1.491 (0.661–3.365)	0.3357		
KPS	> 80	1							
	≤ 80	1.185 (0.375–3.742)	0.7730						
Thrombocytosis	Normal	1				1			
	> 400 K	2.494 (1.437–4.327)	0.0012			1.941 (0.592–6.363)	0.2734		

## Discussion

TT has recently replaced IT as the mainstay therapy for mRCC, resulting in modest survival benefits with improved PFS despite failing to provide measurable benefits to CSS except in poor-risk patients administered temsirolimus [4], as in the present study, where the CSS were the same between poor- and intermediate-risk mRCC. Our previously published study also showed that TT improved prognoses of mRCC patients compared to IT [7]. In this study, we further stratified mRCC patients in terms of metastasis type, Heng risk model, and TFI. The intermediate and poor risk patients were selected in this study; however, patients belonging to the favorable risk group were excluded because of the characteristics of SM, for which no cases belong to the favorable risk group.

The results of our study showed that the intermediate-risk group had significantly better PFS and CSS than the poor-risk group. We also observed that, as compared with IT, TT significantly lengthened PFS, but not CSS (Fig. 1). In particular, a significant difference in CSS was observed in neither the SM subgroup (TT vs. IT: 9.6 vs. 9.1 months) nor the MM subgroup (TT vs IT: 20.1 vs. 25.2 months; *p* > 0.05; Fig. 1b and d). Nonetheless, our results provide some statistically nonsignificant indications that IT may be associated with better CSS than TT: in both the SM and MM subgroups (Fig. 1b and d), comparison of the tails of the IT and TT curves for CSS show that the former had longer durable responses without cancer-specific deaths. Overall, our results suggest that TT was significantly better for PFS, while IT might be more suitable for extending CSS. However, the survival benefit from IT should be considered carefully, because the IT group included patients who benefited from TT (9.5%, *N* = 12), and their follow-up periods were significantly longer than those of TT patients (data not shown). Finally, only SM patients with TFIs < 1 year and MM patients with TFIs ≥1 year had significantly prolonged PFS when treated with TT compared to IT in the intermediate-risk group. Other subgroups did not show any significant survival differences according to treatment.

In previous large-scale IT and TT studies, Motzer et al. [16] and Naito et al. [17] showed median CSS of 10.0 and 21.5 months following cytokine treatment, respectively; the survival times were 14.0 and 29.5 months for their intermediate-risk (according to the Memorial Sloan Kettering Cancer Center [MSKCC] criteria] groups, and 5.0 and 9.8 months for their MSKCC poor-risk groups, respectively [18]. Our study had similar median CSS results, with respectively 13.9 and 25.2 months for the intermediate-risk and 5.6 and 10.2 months for the poor-risk SM and MM groups. Some differences were expected owing to the enrolled patients’ ethnic group, rate of nephrectomy (Motzer: 80.5%, Naito: 55%, and our study: 56.5%), and the different cytokine-treatment regimens and primary and metastatic tumor burdens. The outcomes of TT in our study were similar to, or slightly poorer than, previous phase 3 trials [7, 14]. The different outcomes between this study and previous studies were attributed to the different characteristics of the enrolled patients, including the absence of favorable-risk groups in our study.

To our knowledge, no studies have directly compared prognostic outcomes according to metastasis type outside of metastatic site-specific comparison studies and case reports [19–24]. Although tumor cells’ pathophysiologies, activities, and burdens differ between SM and MM, the systemic treatment protocol for these cancers remain the same. Previous studies and ours showed different pathophysiologic activities of tumor cells as identified by immunohistochemical staining of multiple tissue markers that are closely related to the TT and immune responses [15, 19]. The absence of a primary renal tumor burden post-nephrectomy influences prognoses following both IT and TT [25, 26].

Our study classified mRCC patients according to the Heng risk criteria, TFI, metastatic types, and systemic therapies to determine the influence of each on prognoses. Prognostic comparisons according to the Heng risk groups showed a significant difference in CSS between SM (13.9 months) and MM (25.2 months) in the intermediate-risk group (*p* = 0.010), but not in PFS. The poor-risk group also showed no prognostic differences according to metastatic types. CSS rates were better in MM than in SM patients because the metastatic tumor burden was less in the former, and the tumor metabolic activity was different from those with SM. When systemic therapies were compared, only the PFS in the intermediate-risk group showed a significant improvement with TT over IT in both the SM (6.1 vs. 2.6 months) and MM (10.2 vs. 4.1 months) groups, respectively. One interesting finding was that receipt of IT was associated with nonsignificantly better PFS and CSS rates for poor-risk patients in both the SM and MM subgroups (*p* > 0.05, Fig. 1c and d). When considering TFI, TT significantly improved PFS in SM group patients with TFIs < 1 year and in MM group patients with TFIs ≥1 year; these findings were true regardless of metastatic types. In addition, because of multicollinearity between TFI and metastatic type (SM vs. MM), metastatic type was not a significant prognostic factor in the multivariable analysis, even though it had been significantly prognostic in the univariable analysis.

This study had some inherent limitations owing to its retrospective design, the small number of poor-risk patients, the exclusion of patients with medical records that were incomplete because of loss to follow-up, and the different follow-up periods in the compared subgroups. Most metastatic lesions that were diagnosed pathologically might not accurately represent the whole disease, especially when comparing the primary and various metastatic lesions. Further studies that include tissue analysis, gene sequencing, and clinicopathological data are warranted to identify the therapeutic benefit of systemic therapies on MM and SM patients. Prospective data collection and different statistical analyses may help to address the possibility that selection bias and other biases could have affected the comparisons that were made in the present study. For example, the use of propensity score matching might allow more rigorous comparisons of outcomes after IT and TT, as well as of the prognostic implications of SM and MM mRCC. There was a difficulty in applying propensity score matching in this study. Since some of the variables were unknown, the size of the after matching set will be smaller. It is difficult to analyze each sub-groups (SM/MM, risk group, Treatment interval) after matching because IT and TT survival is identified in all subgroups of patients. Our study is only the second to compare prognoses of SM and MM patients treated with systemic TTs.

## Conclusions

Our study showed that dividing patients into additional subcategories improved the prediction of therapeutic outcomes. We found that patients with intermediate-risk mRCC treated with TT had significantly better PFS rates than those treated with IT among patients with SM and TFIs < 1 year and patients with MM with TFIs ≥1 year.

## Additional files

Additional file 1: **Table S1.** The Cox proportional hazards model of progression-free survival and cancer-specific survival of prognostic risk factors. **Figure S1.** Flow-chart of patients included in analysis. **Figure S2.** Comparison of Kaplan-Meier curves of progression-free survival and cancer-specific survival of metastatic renal cell carcinoma patients according to the metastatic types and risks according to the Heng criteria. **Figure S3.** Comparison of Kaplan-Meier curves of progression-free survival and cancer-specific survival of metastatic renal cell carcinoma patients with synchronous metastases and metachronous metastases according to the Heng criteria risk groups. (DOCX 97 kb)

## Data Availability

All data generated or analyzed during this study are included in this published article [and its supplementary information files].

## Abbreviations

(m)RCC: (metastatic) renal cell carcinoma
CSS: Cancer-specific survival
FU: Fluorouracil
IFN: Interferon
IL: Interleukin
IT: Immunotherapy
MM: Metachronous mRCC
OS: Overall survival
PFS: Progression-free survival
SM: Synchronous mRCC
TFI: Treatment-free interval
TT: Targeted therapy
