# Supplementary material for: Survival of patients receiving systematic therapy for metachronous or synchronous metastatic renal cell carcinoma: a retrospective analysis
Source: BMC Cancer. 2019 Jul 15;19:688. doi: 10.1186/s12885-019-5900-1 (PMC6631605; doi:10.1186/s12885-019-5900-1)

Supplementary Table1. The Cox proportional hazards model of progression-free survival and cancer-specific survival of prognostic risk factors.

|  | | Progression free survival | | | | Cancer specific survival | | | |
| --- | --- | --- | --- | --- | --- | --- | --- | --- | --- |
|  |  | Univariable | | Multivariable | | Univariable | | Multivariable | |
|  |  | Hazard ratio | P-value | Hazard ratio | P-value | Hazard ratio | P-value | Hazard ratio | P-value |
|  |  | (95% CI) |  | (95% CI) |  | (95% CI) |  | (95% CI) |  |
|  |  | N= 214 /EVENT=193 |  | N=207 /EVENT=187 |  | N=214 /EVENT=182 |  | N=202 /EVENT=171 |  |
| metastatic type | MM | 1 |  |  |  | 1 |  |  |  |
|  | SM | 1.364(1.006-1.849) | 0.0453 |  |  | 1.736(1.267-2.381) | 0.0006 |  |  |
| Therapy | IT | 1 |  | 1 |  | 1 |  |  |  |
|  | TT | 0.558(0.417-0.748) | <.0001 | 0.537(0.399-0.724) | <.0001 | 1.271(0.941-1.716) | 0.1184 |  |  |
| Treatment Free interval | ≥1yr | 1 |  | 1 |  | 1 |  | 1 |  |
|  | <1yr | 1.498(1.079-2.080) | 0.0158 | 1.639(1.171-2.293) | 0.0039 | 1.910(1.365-2.673) | 0.0002 | 2.115(1.484-3.015) | <.0001 |
| Gender | Male | 1 |  |  |  | 1 |  |  |  |
|  | Female | 1.109(0.787-1.562) | 0.5551 |  |  | 1.099(0.784-1.541) | 0.5829 |  |  |
| Age |  | 0.989(0.974-1.003) | 0.1185 |  |  | 0.998(0.983-1.012) | 0.7479 |  |  |
| Anemia | Normal | 1 |  |  |  | 1 |  |  |  |
|  | Hb <13.5(male) /12.0(Female) | 1.324(0.936-1.873) | 0.1126 |  |  | 1.18(0.803-1.734) | 0.3989 |  |  |
| Hypercalcemia | Normal | 1 |  |  |  | 1 |  | 1 |  |
|  | >10mg/dL  or 2.5mmol/L | 1.158(0.762-1.760) | 0.4909 |  |  | 1.543(1.023-2.329) | 0.0386 | 1.788(1.163-2.748) | 0.0081 |
| Neutrophilia | Normal | 1 |  | 1 |  | 1 |  | 1 |  |
|  | <1500  or > 7500 | 1.500(1.012-2.221) | 0.0432 | 1.695(1.141-2.516) | 0.0089 | 1.819(1.218-2.718) | 0.0035 | 1.916(1.247-2.942) | 0.0030 |
| Elevated LDH | Normal | 1 |  |  |  | 1 |  |  |  |
|  | 1.5 X ULN | 1.596(1.101-2.313) | 0.0136 |  |  | 1.535(1.046-2.253) | 0.0287 |  |  |
| KPS | > 80 | 1 |  |  |  | 1 |  |  |  |
|  | ≤ 80 | 2.356(0.871-6.373) | 0.0914 |  |  | 1.433(0.456-4.502) | 0.5383 |  |  |
| Thrombocytosis | Normal | 1 |  |  |  | 1 |  | 1 |  |
|  | >400K | 1.545(0.976-2.445) | 0.0635 |  |  | 2.563(1.563-4.203) | 0.0002 | 1.856(1.092-3.157) | 0.0224 |

Supplementary Figure 1. Flow-chart of patients included in analysis.

Supplementary Figure 2. Comparison of Kaplan-Meier curves of progression-free survival and cancer-specific survival of metastatic renal cell carcinoma patients according to the metastatic types and risks according to the Heng criteria.


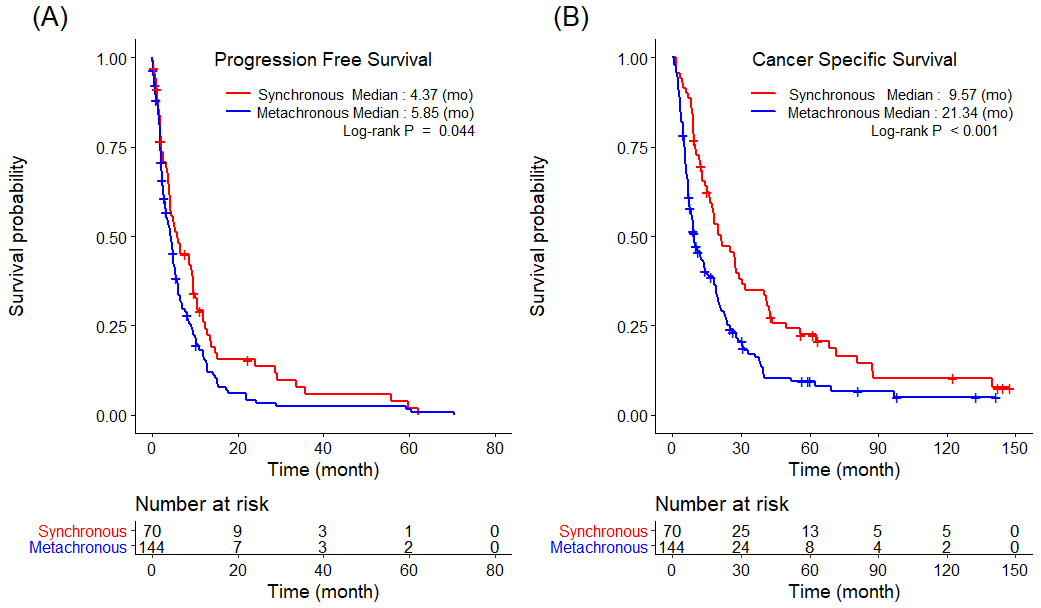


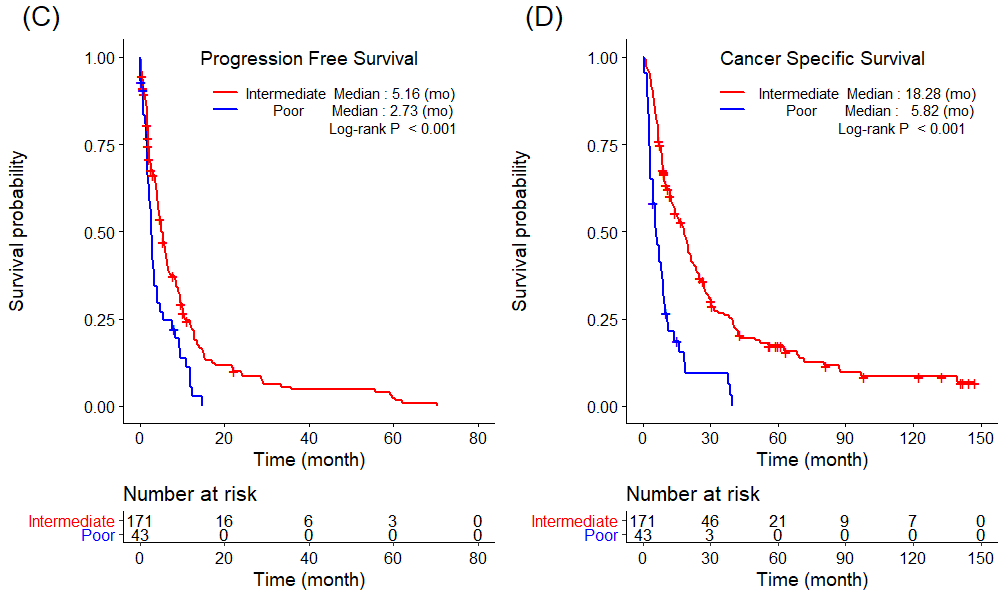


Supplementary Figure 3. Comparison of Kaplan-Meier curves of progression-free survival and cancer-specific survival of metastatic renal cell carcinoma patients with synchronous metastases and metachronous metastases according to the Heng criteria risk groups.


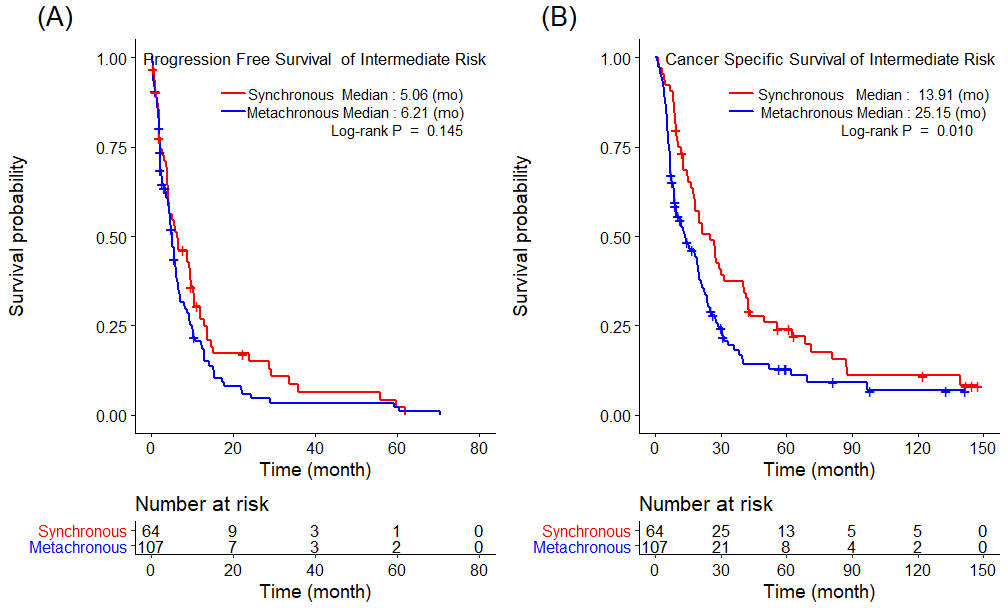


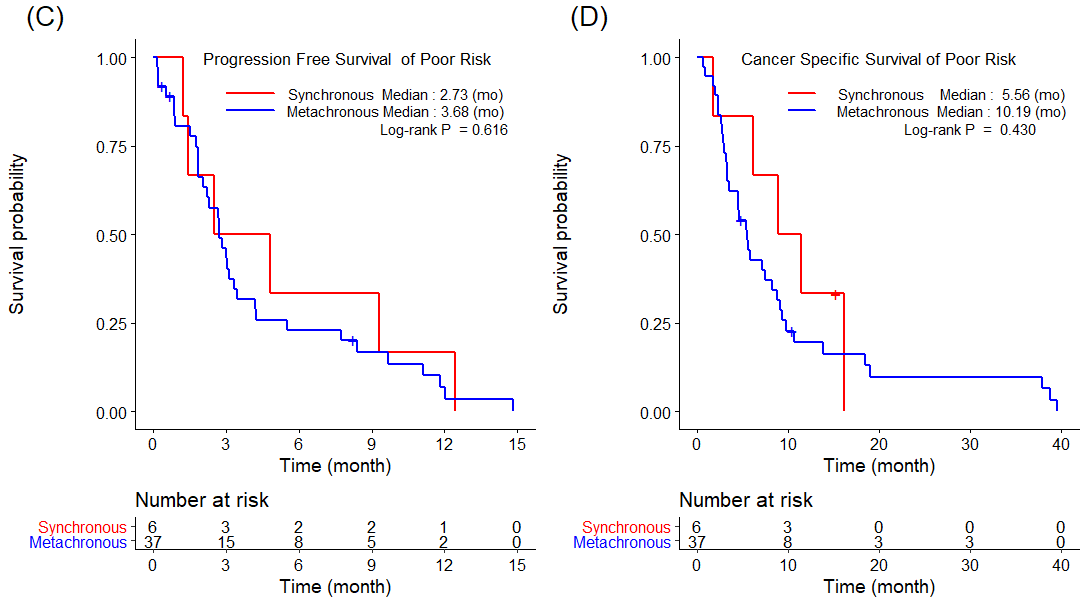

Supplement: Supplementary file 1 — Table S1. The Cox proportional hazards model of progression-free survival and cancer-specific survival of prognostic risk factors. Figure S1. Flow-chart of patients included in analysis. Figure S2. Comparison of Kaplan-Meier curves of progression-free survival and cancer-specific survival of metastatic renal cell carcinoma patients according to the metastatic types and risks according to the Heng criteria. Figure S3. Comparison of Kaplan-Meier curves of progression-free survival and cancer-specific survival of metastatic renal cell carcinoma patients with synchronous metastases and metachronous metastases according to the Heng criteria risk groups. (DOCX 97 kb) [file 12885_2019_5900_MOESM1_ESM.docx]
